# Supplementary material for: Imaging intravoxel vessel size distribution in the brain using susceptibility contrast enhanced MRI
Source: Imaging Neurosci (Camb). 2026 Mar 19;4:IMAG.a.1173. doi: 10.1162/IMAG.a.1173 (PMC13003805; doi:10.1162/IMAG.a.1173)
Supplement: Supplementary Material [file IMAG.a.1173_supp.pdf]

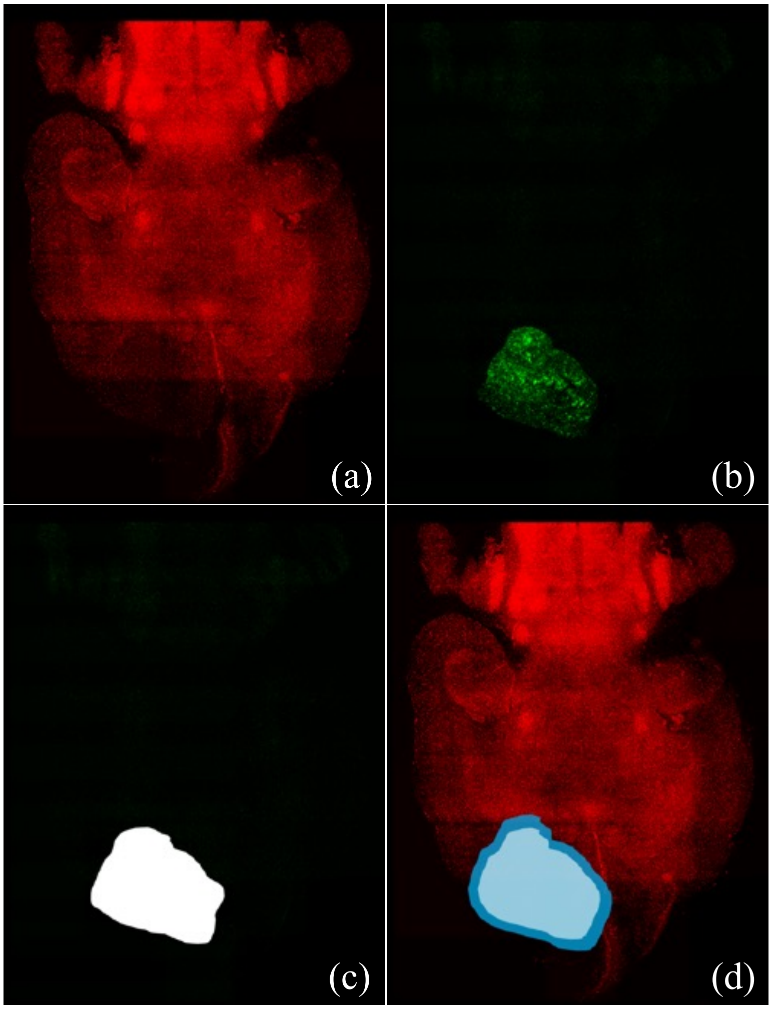


Figure S1. Different steps of Tumor VOI selection. (a,b) Axial slice from the LSFM image of the vasculature (a) and tumor cell nuclei (b) of a rat brain inoculated with GBM10 patient-derived xenograft tumor. (c) The tumor mask (white) manually drawn on (b) using 3D Slicer. (d) The tumor mask is manually enlarged (blue) to cover the peritumoral region and overlapped on (a). The sampled tumor VOIs were completely enclosed within the blue mask region.

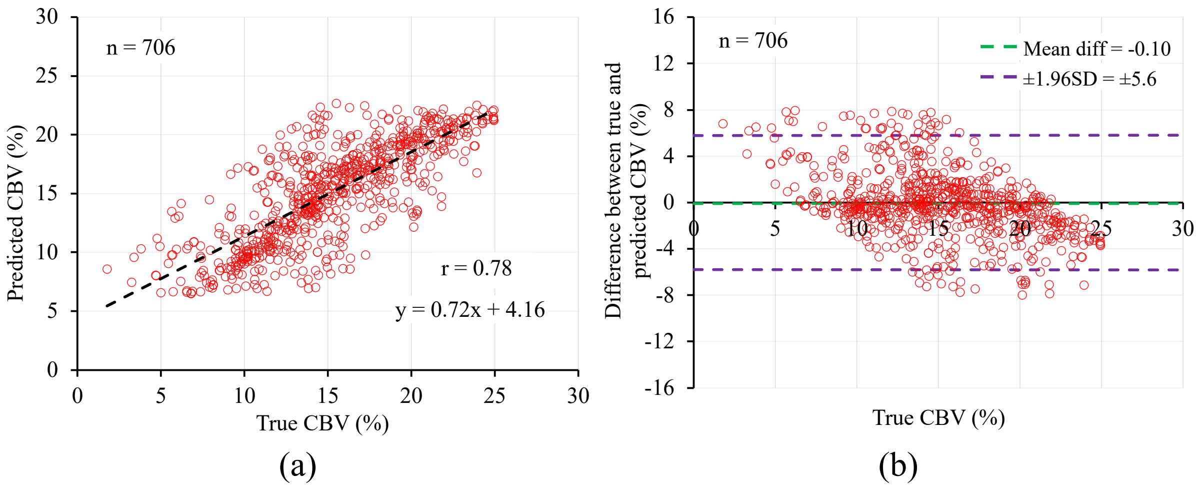


Figure S2. The Pearson correlation (a) and Bland-Altman plots (b) of true and predicted CBV values for the tumor VOIs (n=706).

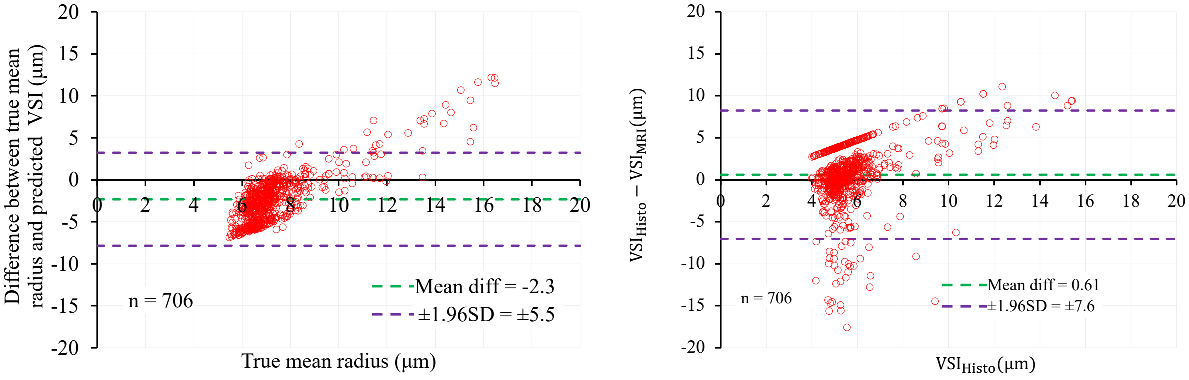


(a) (b)

Figure S3. (a,b) The Bland-Altman plot of difference between true and predicted mean radius (a) and $VSI_{Histo}$ and $VSI_{MRI}$ (b) computed from the tumor VOIs (n=706). The variability of the residuals for true and predicted mean radius values was lower than the residuals for $VSI_{Histo}$ and $VSI_{MRI}$.
